# Functional results and unfavorable events after treatment of proximal humerus fractures using a new locking plate system

**DOI:** 10.1186/s12891-023-06176-5

**Published:** 2023-01-24

**Authors:** Michael Kimmeyer, Jonas Schmalzl, Verena Rentschler, Malik Jessen, Christian Gerhardt, Lars-Johannes Lehmann

**Affiliations:** 1 Department of Traumatology, Hand Surgery and Sports Medicine, ViDia Clinics Karlsruhe, Steinhaeuserstr. 18, 76135 Karlsruhe, Germany; 2 Department of Trauma, Hand, Plastic and Reconstructive Surgery, University Hospital Wuerzburg, Oberduerrbacher Str. 6, 97080 Wuerzburg, Germany; 3 Department of Trauma Surgery, University Clinic Rechts Der Isar, Technical University Munich, Ismaninger Str. 22, D-81675 Munich, Germany

**Keywords:** Proximal humerus fractures, Angle-stable osteosynthesis, Polyetheretherketone, Open reduction internal fixation, Adverse event

## Abstract

**Background:**

Proximal humerus fractures are often treated with a fixed-angle titanium plate osteosynthesis. Recently, plates made of alternative materials such as carbon fibre-reinforced polyetheretherketone (CFR-PEEK) have been introduced. This study presents the postoperative results of patients treated with a CFR-PEEK plate.

**Methods:**

Patients with proximal humerus fractures treated with a CFR-PEEK plate (PEEKPower™ Humeral Fracture Plate (HFP)) were included. In follow-up examination, age and gender adjusted Constant-Murley Score (ACS), Subjective Shoulder Value (SSV), Quick Disabilities of the Arm, Shoulder and Hand Score (QDASH) and pain score (Visual Analog Scale (VAS)) were analyzed. General condition at follow-up was measured by European Quality of Life 5 Dimensions 3 Level Version (EQ-5D-3L). Range of motion was recorded. In addition, radiographs at follow-up, unfavorable events and revision rate were analyzed.

**Results:**

In total, 98 patients (66.0 ± 13.2 years, 74 females, 24 males) were reexamined. Mean follow-up was 27.6 ± 13.2 months. There were 15 2-part, 28 3-part and 55 4-part fractures. The functional scores showed good results: SSV 83.3 ± 15.6%, QDASH 13.1 ± 17.0 and ACS 80.4 ± 16.0. A 4-part-fracture, head split component, nonanatomic head shaft reposition and preoperative radiological signs of osteoarthritis were significant negative predictors for poorer clinical scores. Unfavourable events were observed in 27 patients (27.6%). Revision surgery was performed in 8 (8.2%) patients. Risk factors for an unfavourable event were female gender, age of 50 years and older, diabetes, affected dominant hand, 4-part fracture, head split and preoperative radiological signs of osteoarthritis.

**Conclusion:**

There are several advantages of the CFR-PEEK plate (PEEKPower™ Humeral Fracture Plate (HFP)) such as the polyaxial screw placement and higher stability of locking screws. In summary, the CFR-PEEK plate osteosynthesis is a good alternative with comparable clinical results and some biomechanical advantages. Proximal humerus fractures show good clinical results after treatment with a CFR-PEEK plate. The revision rate and the risk of unfavorable events are not increased compared to conventional titanium plate osteosynthesis.

**Level of evidence:**

IV

## Background

Proximal humerus fractures account for 4 to 6% of all fractures of the skeleton, are more common in elderly women and the majority can be treated conservatively [8, 30]. Dislocated proximal humerus fractures are often treated surgically. A great variety of options is available for the fixation of these fractures including osteosutures, percutaneous Kirschner wire fixation, intramedullary nailing, locking plate osteosynthesis, hemiarthroplasty and recently reverse shoulder arthroplasty [7, 25, 41]. However, all of them suffer from a persistently substantial rate of mechanical failure as well as a number of other complications, including decrease ROM and functional outcomes. The specific treatment decision is based on fracture morphology, bone-quality and patient-specific criteria (e.g. age, physical activity, comorbidities). The optimal treatment of displaced proximal humerus fractures is still controversial. An open reduction enables a good reconstruction of the fracture, and a high level of stability can be achieved through internal fixation using an angle-stable plate fixation [36]. In most cases, this technique allows early functional rehabilitation, so that an immobilization of the shoulder is not needed. The previously established plates are made of titanium. Recently, new osteosynthetic materials such as carbon fiber-reinforced-polyetheretherketone (CFR-PEEK) plates have been used for the surgical treatment of proximal humerus fractures. The CFR-PEEK plates have a modulus of elasticity that is very similar to that of cortical bone [33]. Biomechanical studies showed several advantages like an increased motion at the bone-implant interface and a higher stability of locking screws in the CFR-PEEK plates [15, 22, 34]. Locking titanium plates have a rigid link between plate and screw, which can lead to failure at the bone-screw interface, especially in osteoporotic bone [26]. In addition, reduced micromotion at the fracture site can lead to impaired fracture healing [9, 10]. Therefore, more elastic fixation techniques such as CFR-PEEK materials create optimal biomechanical properties for a good fracture healing process. Another advantage compared to many conventional titanium plates is the possibility of polyaxial screw placement. The radiolucency of the CFR-PEEK plate allows better intraoperative exposure, repositioning of the fracture and placement of the screws. Furthermore, cold welding does not occur in CFR-PEEK plates.

The aim of this study was to evaluate clinical and functional outcomes, radiographic parameters and complications in patients with proximal humerus fractures treated with CFR-PEEK plates.

## Material and methods

This study was a retrospective case series. The patients were operated in a regional trauma center, which is also a certified center of shoulder surgery. The surgeries took place between January 2017 and October 2020. The data were only collected after a signed declaration of consent. A positive vote from the responsible ethics committee is available.

### Study population

Patients with proximal humerus fractures treated with a CFR-PEEK plate (PEEKPower™ Humeral Fracture Plate (HFP), Arthrex®, Naples, United States of America) were reexamined. Included were all patients with the following criteria:

• 2-, 3- and 4-part proximal humerus fracture
• open reduction and internal fixation with a CFR-PEEKPower™ Humeral Fracture Plate
• surgery within 14 days after trauma
• patient aged 18 or older
• minimum follow-up of 1 year
• existing follow-up radiographs
• written informed consent

All patients with multiple fractures of the affected extremity and glenohumeral joint dislocations with fractures were excluded. In addition, patients were excluded if they had a pathologic fracture, or preexisting symptomatic osteoarthritis of the glenohumeral joint. In addition, patients were excluded who, for medical reasons, could not attend a follow-up examination or who could not be contacted. If the inclusion criteria were not met, the patients were not considered.

### Surgical indication and treatment

The specific treatment decision was based on fracture morphology, bone-quality and patient-specific criteria (e.g. age, physical activity, comorbidities). The risk of a fracture sequelae of the proximal humerus according to Boileau was estimated taking into account the criteria according to Hertel [16]. If the fracture could not be reconstructed or if the surgeon considered it was fraught with complications, a primary endoprosthetic joint replacement was performed. The surgery was performed under general or regional anesthesia using an interscalene plexus block. Patients were positioned in beach chair position and a deltopectoral approach was used. Depending on the fracture morphology, a 3 or 5-hole PEEKPower™ Humeral Fracture Plate (HFP) with soft bone locking screws (3.5 or 4 mm) was used. Depending on the situation, suture cerclage of the greater and/or lesser tuberosity (FiberWire®, Arthrex®, Naples, United States of America), additional screw osteosynthesis of the lesser tuberosity, and tenotomy or tenodesis of the long head of the biceps tendon were performed. Depending on fracture morphology and the individual decision of the surgeon, follow-up treatment included an early functional therapy or immobilization in a shoulder abduction splint for 3 weeks with subsequent passive mobilization of the shoulder. In case of a postoperative immobilization of 3 weeks, patients were allowed to start active movements after 6 weeks.

### Functional outcomes and clinical scores

**Fig. 1:**
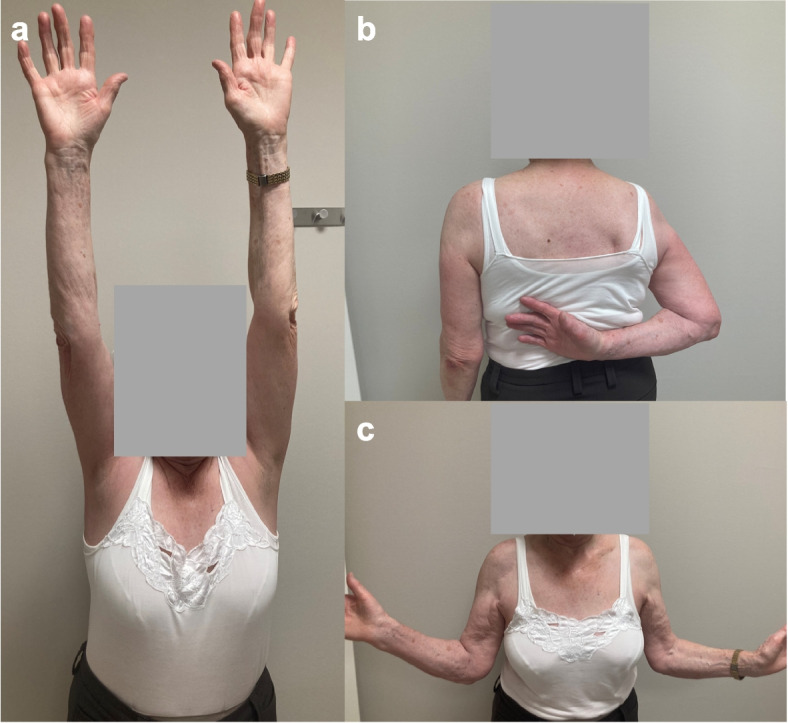
Patient at follow-up after proximal humerus fracture. Testing the range of motion of an 87-year-old female patient at follow-up 3.5 years after a 3-part proximal humerus fracture of the right side treated with CFR-PEEK plate osteosynthesis: a) forward flexion, b) internal rotation, c) external rotation. (CFR-PEEK = carbon fibre-reinforced polyetheretherketone)

At follow-up, active and passive range of motion of the shoulder (abduction, flexion, external rotation, internal rotation) were evaluated (Fig. 1). The isometric abduction strength was measured in 90° in the scapular plane [17]. Two measurements were made per side and the mean value was calculated. Several specific shoulder scores were evaluated: age and gender adjusted CS (ACS), Subjective Shoulder Value (SSV), Quick Disabilities of the Arm, Shoulder and Hand Score (QDASH) and Visual Analog Scale (VAS). General condition at follow-up was measured by European Quality of Life 5 Dimensions 3 Level Version (EQ-5D-3L) and European Quality Visual Analog Scale (EQ-VAS).

### Radiological outcomes

**Fig. 2:**
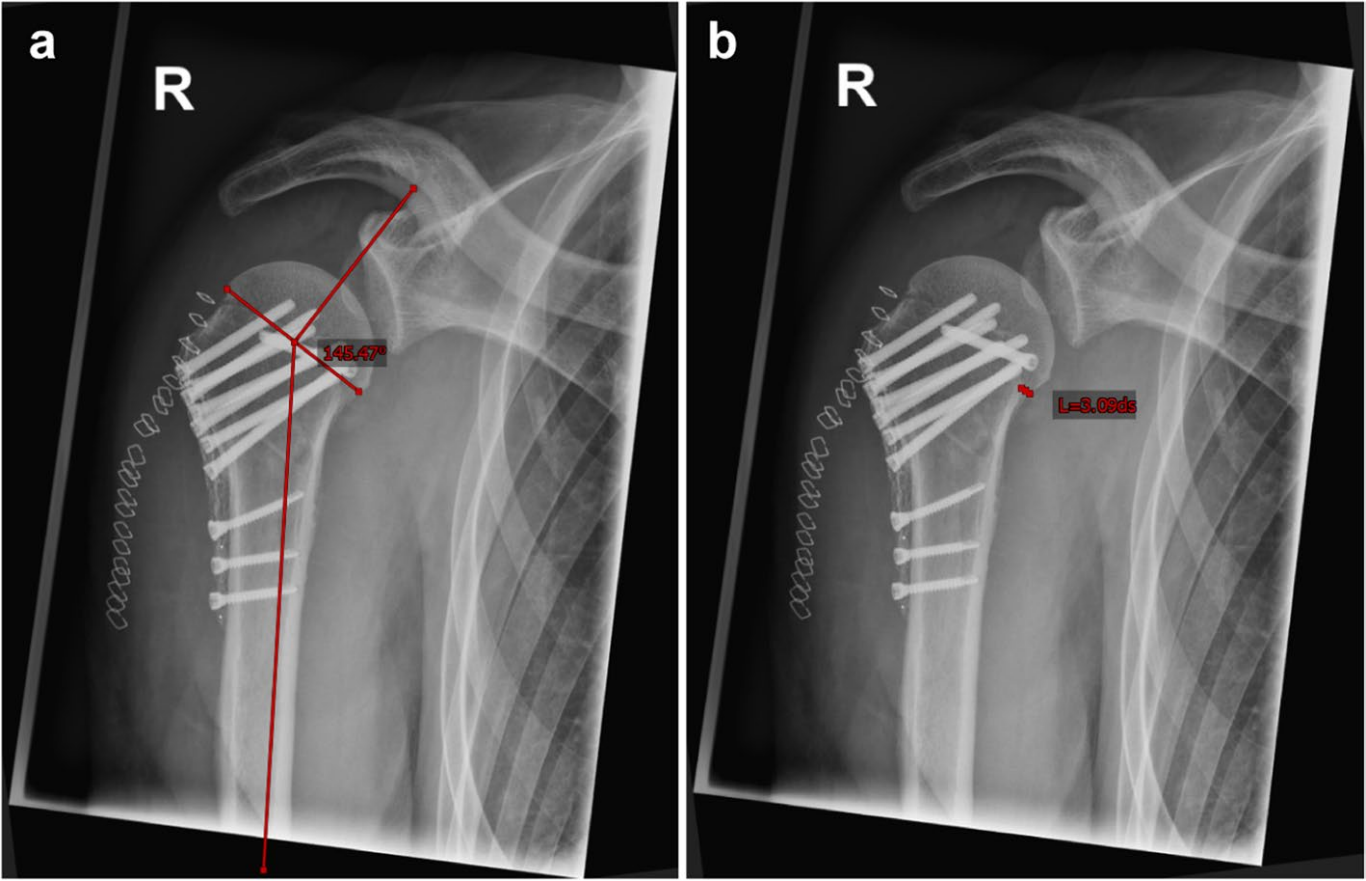
Radiological measurements. Postoperative radiological measurements: radiographs in ap view with a) neck shaft angulation, b) head shaft distance (ap = anteroposterior)

**Fig. 3:**
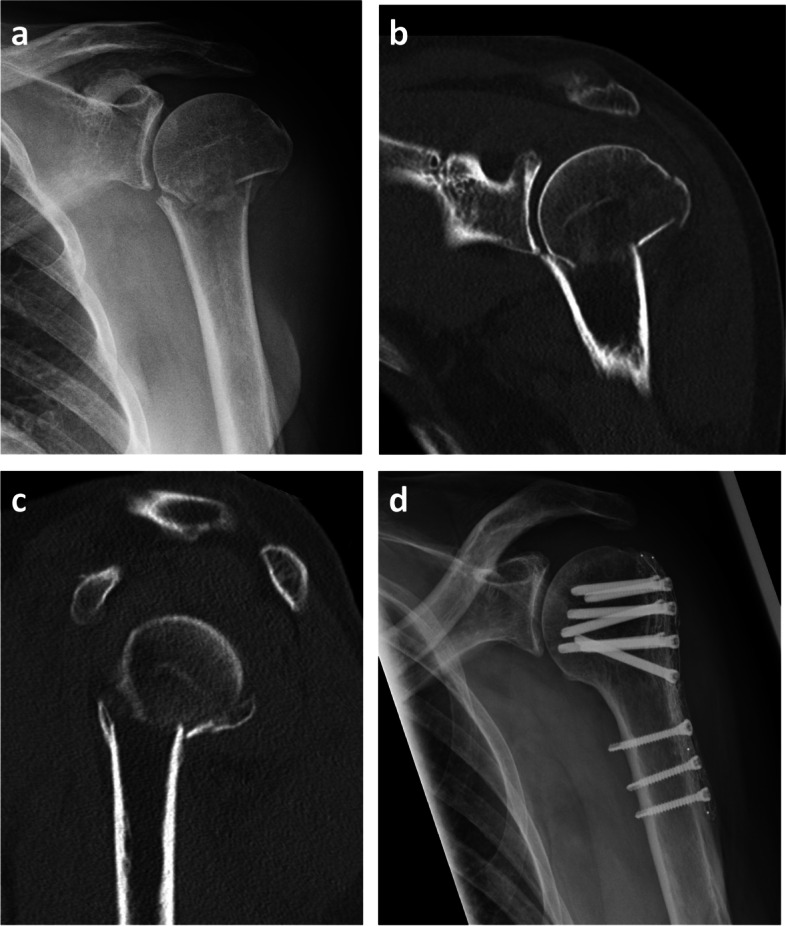
Radiological imaging of a 3-part proximal humerus fracture 69-year-old female patient with 3-part proximal humerus fracture treated with CFR-PEEK plate osteosynthesis; preoperative imaging: **a**) radiograph in ap view, **b**) CT scan in coronal plane, **c**) CT scan in sagittal plane; **d**) postoperative radiograph in lateral view. (CFR-PEEK = carbon fibre-reinforced polyetheretherketone, CT = computed tomography)

Preoperatively performed computed tomography (CT) scans as well as radiographs (true anterior posterior (ap) view, lateral (Y) view) two days postoperatively and at follow-up were evaluated. In preoperative CT scans the morphology of the fracture (Neer), the grade of glenohumeral osteoarthritis (Samilson-Prieto) and the morphology of the glenoid (Walch) were analyzed [28, 31, 40]. According to the AO classification, a head split component was defined when an articular surface fracture was present [27]. In postoperative radiographs, the neck shaft angulation (NSA) was measured (Fig. 2). In addition, dislocation of the greater tuberosity, head shaft distance and intraoperative screw placement were analyzed. At follow-up examination, further radiological parameters were assessed: screw or plate breakage or dislocation, pseudarthrosis, osteonecrosis of the humeral head. The radiological images were assessed by two surgeons and a consensus was reached in the event of differing evaluation. Figure 3 shows pre- and postoperative imaging of a 69-year-old female patient with a 3-part fracture of the proximal humerus.

### Unfavorable events

Based on a recently published literature research by Alispahic et al. the term “unfavorable events” (synonym: complication, adverse events) was used in our study [2]. The unfavorable events were divided into several groups as shown in Table 2. For some of the radiological parameters, the corresponding cut-off values are given. Fracture reduction was measured as anatomic and nonanatomic [11]. Nonanatomic reduction was classified as varus malalignment (NSA < 110°) and valgus malalignment (NSA > 150°) [1, 20, 35]. A malreduction and secondary dislocation of the greater tuberosity or of the head shaft distance are defined from a displacement of more than 5 mm [35]. A secondary osteoarthritis was defined as a complication if the glenohumeral osteoarthritis (classified by Samilson-Prieto) was progressive and painful. A shoulder stiffness was defined as a global limitation of the range of motion with less than 100° in flexion, less than 10° in external rotation and less than L5 level in internal rotation [19]. Pseudoparalysis of the shoulder was defined as active flexion less than 90° with free passive motion [39]. Removal of the CFR-PEEK plate at patient’s request was not rated as a revision, as this is an expected follow-up operation. A plate removal due to clinical symptoms such as restricted mobility and severe implant irritations was recorded as an implant-related unfavorable event.

### Statistical evaluation

The patients were divided into age groups (< 50 years, 50 – 70 years, > 70 years). The proximal humeral fractures were classified according to the fracture morphology (Neer: 2-part, 3-part, 4-part; head split component: yes or no; osteoarthritis: yes or no; head shaft malreduction: yes or no). The ACS was ranked in groups according to the clinical outcome: “very good” (86–100), “good” (71–85), “fair” (56–70), “poor” (< 56) [3]. There is no established minimal clinically important difference (MCID) for the CS and QDASH, but 10 points are considered appropriate [42].

Statistical analyses were performed using SPSS® software (version 28.0; IBM®, Armonk, United States of America). The nominal variables such as gender and fracture type were summarized as percentages. The arithmetic mean and its standard deviation were used for descriptive statistics. Continuous and ordinal variables were grouped with medians and ranges. Shapiro–Wilk test was used to test the normality of the variables. Wilcoxon-Mann–Whitney test (U test) was used for quantitative variables based on distribution normality. For testing the association of two ordinal variables, the Pearson’s chi-squared test was calculated. The Odds’ ratio was performed to quantify the association of qualitative variables. For exploratory statistics, the level of significance was set for *p* < 0.05.

## Results

### Study population

In this study, 98 patients (female = 74 (75.5%), male = 24 (24.5%) were included. A total of 148 patients were treated with CFR-PEEK plates during the defined period. Loss to follow-up was 33.8%. Patients could not be reexamined due to unavailability (3), disability or illness (16) or death (8). 6 patients could not participate because they lived too far away. 9 patients were satisfied with the situation and were not available for a follow-up examination. In addition, 8 patients were not available due to the COVID-19 pandemic.

Mean follow-up time was 27.6 ± 13.2 months. The mean age at follow-up was 66.0 ± 13.2 years. 8 patients were under 50 years, 47 between 50 and 70 years und 43 patients were more than 70 years old. The mean body mass index was 25.5 ± 4.1 kg/m^2^. A total of 8 patients reported regular cigarette consumption. A known diabetes mellitus was reported by 10 patients. In 46 patients, the dominant side was affected. The detailed results are shown in Table 1.

**Table 1:** Study population with clinical and functional outcome parameters and scores according to the number of fracture parts of proximal humeral fractures (*n* = number, SD = standard deviation, BMI = body mass index, SSV = Subjective Shoulder Value, VAS = Visual Analog Scale, QDASH = Quick Disability Arm, Shoulder and Hand Score, CS = Constant-Murley Score, ACS = age and sex corrected Constant-Murley Score, adl = activities of daily living, fu = follow-up, ROM = range of motion)

		**study population**		**number of dislocated fracture parts**					
				**2**		**3**		**4**	
**n**		**98**		**15**		**28**		**55**	
**gender** (n, %)	female	**74**	75.5%	**10**	66.7%	**22**	78.6%	**42**	76.4%
	male	**24**	24.5%	**5**	33.3%	**6**	21.4%	**13**	23.6%
**age** (Mean, SD)		**66,0**	± 13.2	**57.7**	± 18.4	**65.2**	± 12.2	**68.7**	± 11.0
**age group** (n, %)	< 50	**8**	8.2%	**4**	26.7%	**2**	7.1%	**2**	3.6%
	50—70	**47**	48.0%	**6**	40.0%	**13**	46.4%	**28**	50.9%
	> 70	**43**	43.9%	**5**	33.3%	**13**	46.4%	**25**	45.5%
**months surgery to fu** (Mean, SD)		**27.6**	± 13.2	**25.3**	± 11.9	**30.8**	± 15.9	**26.7**	± 11.9
**body mass index** (Mean, SD)		**25.5**	± 4,1	**25.4**	± 4.4	**25.2**	± 3.6	**25.7**	± 4.3
**smoking** (n, %)	yes	**8**	8.2%	**1**	6.7%	**3**	10.7%	**4**	7.3%
	no	**90**	91.8%	**14**	93.3%	**25**	89.3%	**51**	92.7%
**diabetes** (n, %)	yes	**10**	10.2%	**0**	0.0%	**5**	17.9%	**5**	9.1%
	no	**88**	89.8%	**15**	100.0%	**23**	82.1%	**50**	90.9%
**affected dominant hand** (n, %)	yes	**52**	53.1%	**9**	60.0%	**12**	42.9%	**31**	56.4%
	no	**46**	46.9%	**6**	40.0%	**16**	57.1%	**24**	43.6%
**head split component** (n, %)	yes	**32**	32.7%	**1**	6.7%	**3**	10.7%	**28**	50.9%
	no	**66**	67.3%	**14**	93.3%	**25**	89.3%	**27**	49.1%
**Preoperative osteoarthritis** (n, %)	yes	**27**	27.6%	**2**	13.3%	**9**	32.1%	**16**	29.1%
	no	**71**	72.4%	**13**	86.7%	**19**	67.9%	**39**	70.9%
**SSV** (Mean, SD)		**83.3**	± 15.6	**85.6**	± 14.5	**91.1**	± 8.4	**78.7**	± 17.2
**VAS** (Mean, SD)		**1.2**	± 1.9	**1,2**	± 1.9	**0.5**	± 0.8	**1,6**	± 2.1
**QDASH** (Mean, SD)		**13.1**	± 17.0	**13.8**	± 24.1	**4,9**	± 5.1	**17.0**	± 17.4
**CS** (Mean, SD)	ACS	**80.4**	± 16.0	**88.7**	± 8.3	**88.3**	± 8.9	**74.2**	± 17.7
	pain	**13.5**	± 2.3	**13.9**	± 1.9	**14.5**	± 0.9	**12.8**	± 2.6
	adl	**17.5**	± 3.4	**18.3**	± 2.7	**19.3**	± 1.5	**16.3**	± 3.8
	rom	**33.5**	± 8.2	**38.3**	± 3.1	**36.7**	± 5.2	**30.6**	± 9.1
	strength	**13.1**	± 5.9	**16.3**	± 5.6	**14.9**	± 4.2	**11.4**	± 6.2
**active ROM** (Mean, SD)	abduction	**146.0**	± 36.6	**165.3**	± 17.3	**161.4**	± 25.5	**132.9**	± 40.0
	flexion	**146.6**	± 34.9	**163.0**	± 19.8	**163.2**	± 22.8	**133.7**	± 38.0
	external rotation	**47.5**	± 22.1	**59.3**	± 19.1	**53.4**	± 19.2	**41.3**	± 22.4

In 92 patients, a 3-hole CFR-PEEK plate and in 6 patients a 5-hole plate was utilized. In 22 patients, an additional screw for the lesser tuberosity was used. Suture cerclage of the tuberosities was performed in 7 (46.7%) 2-part fractures and in every 3- and 4-part-fractures. Tenotomy or tenodesis of the long biceps tendon was performed in 16 (16.3%) and 7 (7.1%) cases, respectively. Regarding postoperative follow-up, early functional mobilization was performed in most patients. In all 2-part fractures was recommend in all patients. In 3- and 4-part fractures, the restrictive protocol was recommended in 14 (16.9%) patients.

### Clinical and functional outcome and radiological results

The included patients were in good general condition. EQ-5D-3L was 0.97 ± 0.06 and EQ-VAS was 81.5 ± 15.9. SSV was 83.3 ± 15.6%, VAS was 1.2 ± 1.9, ACS was 80.4 ± 16.0 and QDASH was 13.1 ± 17.0. The results of active range of motion of the affected arm were as follows: abduction 146°, flexion 147°, external rotation 48°. 92.9% of patients achieved internal rotation of at least lumbar vertebrae 5. In addition to the clinical and functional outcome, Table 1 shows the results according to the Neer classification.

Based on the radiograph at the time of trauma, 27 patients had osteoarthritic joint abnormalities. According to the Samilson-Prieto classification, 23 cases had osteoarthritis grade 1 and 4 cases had osteoarthritis grade 2. The patients with osteoarthritic joint abnormalities were significantly older (75.6 ± 8.7 vs. 62.3 ± 12.8 years (*p* < 0.001). Body mass index, EQ-5D-3L and EQ-VAS showed no significant differences. There were significant differences in functional results between those with and those without osteoarthritis in CS rom 31.8 ± 8.3 vs. 34.2 ± 8.1 (*p* = 0047) and CS strength 11.1 ± 4.9 vs. 13.9 ± 6.1 (*p* = 0.011).

A head split component was found in 32 (32.7%) patients. No differences were seen in the epidemiologic parameters. Several significant differences between head split group and no head split group were seen: SSV 79.1 ± 16.0% vs. 85.4 ± 15.2% (*p* = 0.033), QDASH 15.6 ± 14.2 vs. 11.8 ± 18.1 (*p* = 0.027), ACS 75.8 ± 14.8 vs. 82.7 ± 16.2 (*p* = 0.005), active abduction 137.3° ± 35.2° vs. 150.2° ± 36.7° (p = 0.008), active flexion 135.8° ± 37.0° vs. 151.9° ± 32.9° (*p* = 0.004), external rotation 40.9° ± 22.2° vs. 50.7° ± 21.5° (*p* = 0.034).

According to head shaft malreduction, significantly better functional results were shown for a malreduction less than 5 mm: SSV 85.8 ± 14.3% vs. 65.8 ± 14.0% (*p* < 0.001), QDASH 11.9 ± 16.9 vs. 21.6 ± 15.9 (*p* = 0.006), ACS 82.8 ± 14.4 vs. 63.9 ± 18.1 (*p* < 0.001), active abduction 151.9 ± 31.4° vs. 103.8 ± 44.2° (*p* < 0.001), active flexion 152.0 ± 30.4° vs. 108.3 ± 42.2° (*p* < 0.001) and active external rotation 51.5 ± 20.0° vs. 18.8 ± 13.5° (*p* < 0.001).

### Unfavorable events and revisions

An overview of all unfavorable events of this case series is shown in Table 2. Unfavorable events were observed in a total of 27 (27.6%) patients, with a cumulative total of 48 registered unfavorable events. The most common were osteochondral events (*n* = 37, 75.5%). In total, 4 (8.2%) implant events were noticed in 3 patients: 2 malpositioning of the plate, 1 screw dislocation and 1 plate fracture. Revision surgery was performed in 8 (8.2%) patients. Reverse total shoulder arthroplasty was done in 3 (3.1%) patients (I: necrosis of the humeral head without screw perforation, II: necrosis of the humeral head with screw perforation, III: head shaft malreduction and tuberosity malreduction with head necrosis and screw perforation/ screw dislocation at follow-up). Due to patient’s request and minor restrictions, the CFR-PEEK plate was removed in 11 (11.2%) patients (Neer 2: 2, Neer 3: 6, Neer 4: 3).

**Table 2:** Overview over all unfavorable events with criteria and frequency (*n* = number, RSA = reverse shoulder arthroplasty)

	**criteria**	**Frequency n%**	
**implant events (*n* = 4, 8.2%)**			
malpositioning of the plate	yes or no	**2**	**4.1%**
screw dislocation	yes or no	**1**	**2.0%**
plate fracture	yes or no	**1**	**2.0%**
**osteochondral events (*n* = 37, 75.5%)**			
postoperative malreduction of the fracture	yes: NSA < 110° or > 150°, no: NSA 110–150°	**3**	**6.1%**
head shaft malreduction	yes: > 5 mm, no: < 5 mm	**12**	**24.5%**
greater tuberosity malreduction	yes: > 5 mm, no: < 5 mm	**5**	**10.2%**
postoperative screw perforation	yes or no	**1**	**2.0%**
secondary tuberosity dislocation	yes: > 5 mm, no: < 5 mm	**1**	**2.0%**
tuberosity resorption	yes or no	**1**	**2.0%**
osteonecrosis with screw perforation	yes or no	**5**	**10.2%**
osteonecrosis without screw perforation	yes or no	**7**	**14.3%**
secondary glenohumeral osteoarthritis	yes: painful and progressive, no: not painful and progressive	**2**	**4.1%**
**infection (*n* = 1, 2.0%)**			
late hematogenous infection	yes or no	**1**	**2.0%**
**deep soft tissue events (*n* = 7, 14.3%)**			
operated shoulder stiffness	yes or no	**5**	**10.2%**
persistent shoulder stiffness	yes or no	**2**	**4.1%**
**total unfavorable events**		**49**	**100.0%**
**patients with unfavorable event**		**27**	**27.6%**

Regarding clinical and functional outcomes, ACS, CS range of motion and CS strength and active flexion of the shoulder were significantly decreased in the presence of an unfavorable event. The ACS was 69.7 ± 18.3 (unfavorable event) vs. 84.5 ± 13.0 (no unfavorable event) (*p* < 0.001). The detailed results are shown in Table 3.

**Table 3:** Clinical and functional outcome according to unfavorable events (*n* = number, SD = standard deviation, SSV = Subjective Shoulder value, VAS = visual Analog Scale, QDASH = Quick Disability Arm, Shoulder and Hand Score, CS = Constant-Murley Score, ACS = age and sex corrected Constant-Murley Score, adl = activities of daily living, ROM = range of motion)

		**unfavorable events**						
		**Yes *n* = 27**		**No *n* = 71**		**difference**		***p*-value**
**SSV** (Mean, SD)		**74.6**	± 16.8	**86.6**	± 13.9	**-12.0**	-13.9%	**< 0.001**
**VAS** (Mean, SD)		**1.5**	± 2.2	**1.2**	± 1.7	**0.3**	25.0%	0.535
**QDASH** (Mean, SD)		**16.2**	± 16.8	**11.9**	± 17.0	**4.3**	36.1%	0.123
**CS** (Mean, SD)	ACS	**69.7**	± 18.3	**84.5**	± 13.0	**-14.8**	-17.5%	**< 0.001**
	pain	**12.9**	± 2.8	**13.7**	± 2.0	**-0.8**	-5.8%	0.140
	adl	**15.3**	± 3.9	**18.3**	± 2.7	**-3.0**	-16.4%	**< 0.001**
	rom	**28.4**	± 10.5	**34.5**	± 6.1	**-6.1**	-17.7%	**0.001**
	strength	**9.4**	± 5.6	**14.6**	± 5.5	**-5.2**	-35.6%	**< 0.001**
**active ROM**	abduction	**126.1**	± 45.5	**153.6**	± 30.0	**-27.5**	-17.9%	**0.003**
(Mean, SD)	flexion	**127.2**	± 44.8	**154.0**	± 27.3	**-26.8**	-17.4%	**0.004**
	external rotation	**29.6**	± 23.7	**54.3**	± 17.2	**-24.7**	-45.5%	**< 0.001**

There were several factors influencing the development of an unfavorable event: female gender (Odds’ ratio 2.25), age more than 70 years (Odds’ ratio 1.27), diabetes mellitus (Odds’ Ratio 1.88), affected dominant hand (Odds’ ratio 1.41), 4-part fracture (Odds’ ratio 1.84), head split component (Odds’ ratio 1.64) and preexisting osteoarthritic glenohumeral joint abnormalities (Odds’ ratio 1.47).

## Discussion

### Clinical and functional outcome

A systematic review of Brorson et al. listed several studies analyzing locking plate osteosynthesis in intraarticular fractures of the proximal humerus. The ACS of the included studies ranged from 60 to 88% [5]. Good clinical results were also found in a prospective multicenter study of Brunner et al. (CS: 72). So far, some studies on PEEK plate osteosynthesis for proximal humerus fractures have been published. In a retrospective controlled study, Padolino et al. reexamined a total of 42 patients with proximal humerus fractures (21 titanium plates, 21 CFR-PEEK plates) [29]. They had a follow-up time of 30.7 months. The majority of fractures were 3-part fractures (67%). The CS was improved in the CFR-PEEK group (66.3 versus 63.3). Schliemann et al. reported the clinical outcome of 29 patients after 24 months postoperatively who were treated with a CFR-PEEK plate. The mean CS was 71.3 points, and they concluded that the results were comparable to those achieved with conventional locking plates [32]. In a prospective randomized study, Ziegler et al. compared 37 patients with a CFR-PEEK plate and 39 patients with a conventional titanium plate [43]. Follow-up examinations were 6 weeks, 12 weeks and 6 months postoperatively. The DASH of patients treated with the CFR-PEEK plate was 27.5, whereas the titanium plate group scored 28.5. Instead of the DASH, we determined the QDASH, but both scores seem applicable with similar accuracy [13]. The QDASH achieved good to very good results (13.1) in our study. When comparing the CS of the studies mentioned, good functional results can be achieved after treating proximal humerus fractures using PEEK plate osteosynthesis. Good functional results (CS: 77.6, ACS: 80.4) were also achieved in our study, although the majority were 4-part fractures (56.1%). Compared to 2- and 3-part fractures, the functional outcome was significantly reduced in 4-part fractures. Patients with a head split component also showed significantly poorer functional results. Gavaskar et al. showed that locked plating achieves satisfactory results in simple head split fractures (isolated head split fractures). But in complex head split fractures (associated tuberosity fractures), they have seen a poorer shoulder function and a higher complication rate [12]. In our study, we did not differentiate between simple and complex head split fractures. In addition, in our study population, the mean age was higher (68 vs. 38 years) and higher proportion of women (76% vs. 31% women). Because of these reasons, the results are not directly comparable.

### Unfavorable events and revisions

Although locking plate fixation in proximal humerus fractures is frequently performed, unfavorable events and revision surgery are common. Südkamp et al. evaluated the postoperative results of 187 patients with displaced proximal humerus fractures. They found 34% of complications after locking plate osteosynthesis [37]. In other studies, complication rates of up to 49% were reported [4, 6]. Beside the osteoporotic bone, the rigidity of titanium implants could be reason for these high rates. Compared to conventional titanium plates, the CFR-PEEK plates have a modulus of elasticity that is very similar to that of cortical bone. According to biomechanical studies, fixation of unstable proximal humerus fractures with a CFR-PEEK plate allows an increased motion at the bone-implant interface compared with a titanium locking plate, which might be an advantage [23, 33]. Another biomechanical study showed an equal or higher stability of locking screws in CFR-PEEK plates compared to locking screws in stainless steel plates [15].

There are several clinical studies in the literature that analyzed both the functional results and unfavorable events after angular stable plate fixation of the proximal humerus. In a systematic review, Gupta et al. included 2939 proximal humerus fractures treated with an osteosynthesis. A total of 374 (12.7%) revision surgeries were documented, including 91 (3.1%) implant removals, 35 (1.2%) revisions for hemiarthroplasty and 1 (0.04%) revision for reverse arthroplasty. Moreover, 189 (6.4%) cases of humeral head necrosis were found [14]. In another systematic review, Thanasas et al. summarized 12 studies with a total of 791 patients who were operated on using angle-stable plate osteosynthesis. The following unfavorable events were found: infection (1.9%), nonunion (1.6%), humeral head necrosis (7.9%), hardware failure (0.7%), implant loosening (2.6%), displacement (12.2%), implant perforation (11.6%) and a revision rate of 13.7% [38]. Brunner et al. found a rate of unfavorable events of 35% in a prospective multicenter study [6]. The incidence of implant-related complications was 9%. Proximal plate and screw pullout were observed in 2 (1.3%) patients. In addition, 1 (0.6%) breakage and 1 (0.6%) screw backing out were seen. The most common implant-associated unfavorable event was secondary screw perforation (13, 8.2%). In the comparison study of Padolini et al., they found several unfavorable events like tuberosity resorption (14%), varus or valgus malalignment of the humeral head (10%) and humeral head collapse (5%) [29].

In literature, there are several definitions of undesirable postoperative events such as “complications” or “adverse events”. In a systematic review, Alispahic et al. summarized and sorted all the unfavorable events which were found in studies of proximal humerus fractures. In our study, the unfavorable events are presented in detail and in a differentiated manner, as Alispahic et al. have published [2]. A total of 48 unfavorable events were seen in 27 (27.6%) patients in our study. This rate of patients with unfavorable events was higher than in some of the mentioned studies. One reason could be that there is no clear definition in the literature of what a complication, an adverse event or an acceptable postoperative finding is. In relation to the number of unfavorable events, only a low revision surgery rate (8.2%) was found in our cohort. In addition, despite unfavorable events, patients showed good (SSV, QDASH) and fair (ACS) functional results with low pain level (VAS).

Similar to other studies, preexisting diabetes mellitus, female gender, 4-part-fracture and head split were risk factors for unfavorable events and revision surgeries [21]. In addition to these factors, patient age was also an important factor influencing functional outcome and revision rate in our study. In this patient population, the indication for osteosynthesis should be well considered. In a literature review, Kelly et al. found in elderly patients, that reverse prosthesis was superior to reconstructive treatments in terms of range of motion, patient satisfaction and revision rate [24]. Iacobellis et al. concluded in a case series of 33 patients (mean age 76.6 years) that reverse prosthesis is a suitable treatment option for patients with a 3- or 4-part fracture of the proximal humerus and an age greater than 65 years [18]. These studies show that the decision about treatment must be made on an individual basis and several factors must be taken into account. In this context, the risk for adverse events such as osteosynthesis failure and fracture sequelae must always be evaluated to determine the best therapy.

### Limitations

Several limitations of the presented study have been identified. One weakness is the retrospective study design. Because of the chosen study design, differences in surgical technique (tenodesis vs. teonotomy of the long biceps tendon, suture tracings of the tuberosity) and postoperative treatment (early mobilization vs. restrictive protocol) were noted during the analysis. These differences may affect both functional outcome and the occurrence of adverse events. A prospective study design would have been necessary to eliminate this bias. Another limiting factor was the minimum follow-up period of 12 months. Most unfavorable events, such as head necrosis or implant associated complications occur within the first postoperative year, but potentially later unfavorable events could not be identified. In addition, only a small number of patients were available for follow-up in the clinic, resulting in a high loss to follow-up. One reason could be the pandemic situation due to the COVID-19 infection during the study period. Another limitation is the heterogeneous population. Nevertheless, this study included a representative cohort of 98 patients and reported a comprehensive analysis of functional results and a detailed listing of unfavorable events after proximal humerus fractures treated with a CRF-PEEK plate.

## Conclusion

This study shows that good functional results can be achieved in proximal humerus fractures treated with PEEKPower™ Humeral Fracture Plate. The incidence of unfavorable events is comparable to other studies with titanium locking plates. An increased incidence of implant-related complications could not be observed in this study. In summary, the PEEKPower™ Humeral Fracture Plate is a good alternative to conventional titanium locking plate osteosynthesis with some biomechanical advantages such as the polyaxial screw placement, higher stability of locking screws, no cold-welding and radiolucency. However, long-term follow-up examinations and more clinical studies are necessary to confirm our data.

## Data Availability

The datasets used and/or analysed during the current study are available from the corresponding author on reasonable request as participants of this study did not agree for their data to be shared publicly.

## Abbreviations

ACS: Age and gender adjusted constant-murley score
ap: Anterior posterior
CFR-PEEK: Carbon fibre-reinforced polyetheretherketone
CT: Computed tomography
EQ-5D-3L: European Quality of Life 5 Dimensions 3 Level Version
HFP: Humeral Fracture Plate
MCID: Minimal Clinically Important Difference
NAS: Neck Shaft Angulation
QDASH: Quick Disabilities of the Arm, Shoulder and Hand Score
ROM: Range of motion
SSV: Subjective Shoulder Value
VAS: Visual Analog Scale
